# Developing effective chronic disease interventions in Africa: insights from Ghana and Cameroon

**DOI:** 10.1186/1744-8603-6-6

**Published:** 2010-04-19

**Authors:** Ama de-Graft Aikins, Petra Boynton, Lem L Atanga

**Affiliations:** 1 Department of Social and Developmental Psychology, Faculty of Politics, Psychology, Sociology and International Studies, University of Cambridge, Cambridge, UK; 2 Department of Primary Care and Population Health, University College London, London, UK; 3 Department of African Studies, University of Dschang, Dschang, Cameroon

## Abstract

**Background:**

Africa faces an urgent but 'neglected epidemic' of chronic disease. In some countries stroke, hypertension, diabetes and cancers cause a greater number of adult medical admissions and deaths compared to communicable diseases such as HIV/AIDS or tuberculosis. Experts propose a three-pronged solution consisting of epidemiological surveillance, primary prevention and secondary prevention. In addition, interventions must be implemented through 'multifaceted multi-institutional' strategies that make efficient use of limited economic and human resources. Epidemiological surveillance has been prioritised over primary and secondary prevention. We discuss the challenge of developing effective primary and secondary prevention to tackle Africa's chronic disease epidemic through in-depth case studies of Ghanaian and Cameroonian responses.

**Methods:**

A review of chronic disease research, interventions and policy in Ghana and Cameroon instructed by an applied psychology conceptual framework. Data included published research and grey literature, health policy initiatives and reports, and available information on lay community responses to chronic diseases.

**Results:**

There are fundamental differences between Ghana and Cameroon in terms of 'multi-institutional and multi-faceted responses' to chronic diseases. Ghana does not have a chronic disease policy but has a national health insurance policy that covers drug treatment of some chronic diseases, a culture of patient advocacy for a broad range of chronic conditions and mass media involvement in chronic disease education. Cameroon has a policy on diabetes and hypertension, has established diabetes clinics across the country and provided training to health workers to improve treatment and education, but lacks community and media engagement. In both countries churches provide public education on major chronic diseases. Neither country has conducted systematic evaluation of the impact of interventions on health outcomes and cost-effectiveness.

**Conclusions:**

Both Ghana and Cameroon require a comprehensive and integrative approach to chronic disease intervention that combines structural, community and individual strategies. We outline research and practice gaps and best practice models within and outside Africa that can instruct the development of future interventions.

## Background

Africa faces an urgent but 'neglected epidemic' of chronic disease [1,2]. In many countries disability and death rates due to chronic diseases such as diabetes, hypertension and stroke have accelerated over the last two decades. Affected populations include urban and rural, wealthy and poor, old and young. Africa's chronic disease burden has been strongly attributed to changing behavioural practices (e.g sedentary lifestyles and diets high in saturated fat, salt and sugar), which are linked to structural factors such as industrialization, urbanization and increasing food market globalization [1-4]. It is compounded by weak health systems that are unable to cope with the double burden of infectious and chronic diseases. Experts such as Unwin and colleagues (2001) [5] recommend a three-prong approach to dealing with the burden: (1) epidemiological surveillance; (2) primary prevention (preventing disease in healthy populations); and (3) Secondary prevention (preventing complications & improving quality of life in affected communities). Given the well documented challenges in health systems and health policy, experts emphasise that interventions have to be developed within a 'multifaceted and multi-institutional' framework that makes efficient use of existing economic and human resources [1,6-8].

Of the three recommended intervention strategies, epidemiological surveillance has received the most funding and research attention. National surveys have been conducted on risk factors for chronic disease or on general health but with implications for chronic disease. These include STEP Wise Surveys for NCD risk factor surveillance, Global Youth Tobacco Surveys, Global School Health Surveys, Demographic and Health Surveys, World Health Surveys and the Study of Global Ageing and Adult Health (SAGE). Primary and secondary prevention has been largely neglected (with the exception of community-based interventions in Mauritius [8], Tanzania [8], and South Africa [9]). This neglect is problematic. Unhealthy diets, physical inactivity, tobacco and alcohol use have been identified as the major risk factors for chronic diseases. These risk factors are lifestyle-related and can be prevented. There is strong scientific evidence to suggest that by changing to a 'healthier diet, increasing physical activity and stopping smoking, up to 80% of cases of coronary heart disease, 90% of type 2 diabetes cases, and one-third of cancers can be avoided' [1]. Therefore primary prevention strategies must be at the forefront of the regional fight to reduce prevalence rates. Research suggests that in many countries lay knowledge of the risk factors of diabetes, hypertension and stroke is poor [10-12]. With respect to secondary prevention, morbidity and mortality rates of major chronic diseases are high. In countries like Ghana, Nigeria and Cameroon stroke, hypertension, diabetes and cancers cause a greater number of adult medical admissions and deaths compared to communicable diseases such as HIV/AIDS or tuberculosis. Individuals living with these chronic diseases have poor knowledge of their conditions and how to manage them [13-15]. High rates of disability and premature death are linked to poor knowledge and management as well as poor quality services (especially lack of medicines and medical equipment) and poor health worker knowledge. Urgent calls have been made for improved treatment, management and quality of care [11-15].

In this paper we discuss the challenge of developing effective primary and secondary prevention to tackle Africa's chronic disease epidemic through in-depth case studies of research, intervention and policy responses in Ghana and Cameroon.

## Conceptual Framework

Public health education in many African countries is based on a didactic knowledge-attitude-behaviour (KAB) model. The KAB model which is endorsed by the WHO and has featured strongly in HIV/AIDS education derives from social cognition theories and models in psychology that posit a direct link between individual knowledge, attitudes and behaviour. It promotes the notion that greater and better individual knowledge will lead to desired health behavioural change. Critics argue that the KAB model simplifies the complex psychological relationships between knowledge, attitudes and behaviour. A vast literature on health promotion in the areas of smoking [1,7,16], condom use and HIV prevention [17,18] suggests that while health knowledge and literacy are important, mere dissemination of expert health knowledge to lay communities does not result in attitudinal or behavioural change and may in some instances create confusion and anxiety. The empirical evidence suggests that social, political, economic and cultural factors influence individuals' perceptions and definitions of health and illness, their strategies for dealing with health problems and the resources they choose to use during periods of illness. For example, despite having full knowledge of the dangers of smoking, individuals might smoke because it serves important psychological functions, such as relieving stress or strengthening friendship ties [7,16]. These complex lay perceptions and knowledge have been termed 'alternative rationalities' (of reality and health). Similar complexities are identified in everyday experiences of illness. Health psychologists coined the term 'social logic' to describe the way chronically ill individuals make sense of their illness and management routines by drawing from intersubjective experiences and on a broader repertoire of practical routines aimed at addressing the physiological as well as social dimensions of living with illness [19]. In contrast, health experts draw on 'medical logic' which is informed by a disease centred approach to illness and focuses on a restricted repertoire of practical routines aimed at addressing the physiological dimension of the illness.

Within psychology, two current perspectives on health promotion are useful to evaluating the 'multi-faceted and multi-institutional' responses to Africa's chronic disease burden. Public health psychology discussions have focused on the global chronic disease burden. Hepworth (2004), for instance, makes three important arguments [20]. First, she notes that the rise in preventable chronic diseases 'require a contribution from psychology to address modifiable risk factors such as behaviours related to diet and exercise'. Second she observes that individualistic models of human behaviours, such as the KAB models, do not easily translate to public health problems related to patterns of health and disease, for instance geographical, socio-economic, gender, age and ethnic distributions. Models need to be multi-level, ideally addressing individual, social and structural levels of analyses. Finally, she argues that to achieve these multi-level models of health improvement public health psychology needs to develop a 'strategic framework' or matrix of intra-disciplinary (e.g encompassing health, social and community psychology) and interdisciplinary (e.g encompassing psychology, sociology, medicine and economics) approaches. Hepworth's ideas map onto major discussions on chronic disease prevention that identify three important targets for intervention: (1) the individual; (2) the community; and (3) the social or structural (See Table 1).

**Table 1:** Multifaceted and multi-institutional framework for chronic disease prevention

Level of social organisation	Strategies/Actors	Description and African Examples
Structural	Policy	Targeting specific chronic diseases or risk factors (e.g smoking, alcohol)
	
	Fiscal	Taxes on food, alcohol or tobacco. Subsidies on exercise equipment. *South Africa on tobacco; Zambia on soft drinks*[1]
	
	Industry and private businesses	Working with food industry to lower fat or sugar content of products *Mauritius and the food industry *[8]
	
	International collaboration	Building intellectual, technical and financial capacity through partnerships *Mauritius and Tanzania on the InterHealth Project *[8]

Community	Mass media	Public health education via radio, television and newspapers targeting communities or the nation *South Africa and the Coronary Risk Factor Study *[9]
	
	Voluntary/advocacy organisations	Public education, patient support, lobbying by special interest groups.
	
	Institutions (schools, workplace, churches)	Institution-based interventions on diet, physical activity and smoking
	
	Primary healthcare	Routine advice given by doctors and nurses on major risk factors; quality of care; community outreach services. *South Africa and the Coronary Risk Factor Study *[9]

Individual	Behavioural interventions	Tobacco cessation, increased physical activity and dietary change and promotion of weight loss
	
	Pharmacological interventions	Pharmacological interventions for high risk individuals: e.g combination of aspirin, beta-blockers, angiotensin converting enzyme inhibitors and statins can reduce the risk of recurrent myocardial infarction by 75% [1].

Applied social psychology discussions have centred on the importance of 'a social psychology of participation' for community health development (Campbell and Jovchelovitch, 2001) [21]. 'Participation' has produced different meanings and applied method, however two main approaches are distinguished [22,23]. First, the 'utilitarian' or 'top-down' approach conceptualises participation as technocratic use of groups and communities for legitimating projects. While groups may be instrumentally involved in such projects, they are excluded from decision making and sharing political and economic power. Second, the empowerment model or 'bottom-up' approach views participation as a means of empowering marginalized people to make their own health choices and critically foregrounds as its broader objective socio-political change. Research suggests that neither approach in isolation has yielded sustainable results. The social psychology of participation approach emphasises a multi-level framework that combines the strengths of top-down and bottom-up approaches. Theorists stress that this framework must be underpinned by two considerations. First it is important to 'understand each context in its own right', which means prioritising the 'local context' perspective and experience in development programmes [21]. Second, interventions and evaluations must reflect and legitimise the complex inter-relationship between different knowledge systems, identities and power dynamics within lay communities (e.g social logic), health systems (e.g medical logic) and the policy making world (e.g the ideology of development) [24].

Our conceptual framework is informed by these applied psychology perspectives. They facilitate a critical examination of the ways in which our focal countries are responding to their chronic disease burden in 'multi-faceted and multi-institutional' ways. Informed by Hepworth's (2004) public health psychology approach we ask: (1) what levels of analysis are being addressed in country responses: individual, community or structural?; (2) To what extent is the prevailing research culture multidisciplinary and/or based on the right 'strategic framework'? Using Campbell and Jovchelovitch's (2001) social psychology of participation we identify the groups, communities and institutions engaged in concrete primary and secondary prevention activities and evaluate whether their collective activities constitute top-down, bottom-up or multi-level approaches. We identify the factors enabling or undermining their practices.

## Methods

We present and compare two case studies of Ghanaian and Cameroonian responses to their chronic disease burden. We chose Ghana and Cameroon for conceptual and practical reasons. Many countries recognise their local burden but have no policies or plans. In a minority of countries sufficient political will has been generated to ensure the development and implementation of policies. Ghana belongs to the former category, Cameroon to the latter. In international discussions of model African responses to chronic disease burden, South Africa, Tanzania and Mauritius have featured strongly. There are few discussions on model responses from West Africa. We envisaged that a focus on Ghana and Cameroon would: (1) provide insights for countries with similar socio-economic status and burden levels; and (2) focus attention on challenges and model responses in the West African region. We also chose both countries for practical reasons. Two of the authors have extensive research experience and access to the health research communities in these countries (ADGA in Ghana, LLA in Cameroon). We envisaged that practical knowledge of the focal countries would facilitate access to hard-to-reach but theoretically relevant groups and data. General profiles of Ghana and Cameroon drawn from standardised data [25,26] are presented in Table 2.

**Table 2:** Demographic and Socio-economic statistics of Ghana and Cameroon

	Ghana	Cameroon
Population (2007)	23,461,523	18,532,799

GNI Per Capita (US$)	320	630

Life expectancy	60.01	50.39

% popn living in rural areas	50.72	44.06

% popn living in poverty (<$1 per day)	44.8 (1998-99)	17.1 (2001)

Doctor per 10,000	2	2

Nurse/Midwives per 10,000	9	16

Sources: WDI (2009) [25], WHS (2009) [26]

For our review we were interested in two themes: (1) lay knowledge of the major chronic diseases - hypertension, stroke, diabetes, cancers, asthma, sickle-cell disease and their risk factors; and (2) primary and secondary prevention strategies. Our review was limited to medical and social science research employing a broad range of methods, which provided insights for primary and secondary prevention. Prevalence rates of major chronic diseases and their risk factors were sourced from published papers reporting standardized surveys (WHS, STEPs) and national level surveys (see Table 3) [10-12,27]. A literature search of the PUBMED database was conducted focusing on the following subject headings: "hypertension", "diabetes", "cancers", "asthma" "sickle-cell disease" "obesity", "physical activity", "chronic disease", "chronic disease intervention" "self-help groups" "patient advocacy" and "Ghana" and "Cameroon". We focused on the period 1990 - 2009; the burden of chronic disease became officially recognised by policymakers and in policy documents around the early 1990s for both countries. A manual search was conducted in the *Ghana Medical Journal*, *West African Journal of Medicine *and (its previous version) the *West African Medical Journal*, for additional studies on these themes. We contacted key medical and social science researchers working on our focal chronic diseases in Ghana and Cameroon for published or ongoing studies on chronic disease interventions, as well as knowledge on chronic disease advocacy. For further information on self-help and advocacy groups we identified organisations through our research networks and a snowball process. In Ghana, two further strategies were employed: (1) a manual search of medical and public health conference and workshop proceedings; and (2) Ministry of Health annual reports and Programme of Work reports since 1990.

**Table 3:** Prevalence of chronic diseases and risk factors in Ghana and Cameroon

	Ghana		Cameroon	
	**Male**	**Female**	**Male**	**Female**

*Prevalence of chronic diseases*				

Diabetes prevalence estimates* (no of people with DM aged 20-79 (thousands) (2003)	185.0	149.0	23.9	34.5

IGT prevalence estimates* (no of people with IGT aged 20-79 (thousands) (2003)	564.8	636.3	104.5	56.4

Hypertension prevalence**	33.4 (u) 27 (r)	28.9(u) 27 (r)	25.6	23.1

Stroke deaths*** (age standardised mortality per 100 000 population) (2002)	123	151	133	163

Prevalence of Risk Factors****				

Smoking prevalence	6.4	0.5	8.2	1.0

Alcohol consumption (% life-time abstainers)	51.8	61.3	11	18

Physical Activity (insufficient in last 7 days)	7.8	13.2	-	-

Fruit and Vegetable Intake (insufficient intake)	39.6	38.2	-	-

Overweight prevalence (women)	-	17.2	-	20.6

Obesity prevalence (women)		8.1		8.2

Sources:

*IDF, 2003, cited by Mbanya and Ramiaya (2006) [27]

**Ghana figures based on 2004 survey data from Agyemang (2006) and Agyemang et al (2006); Cameroon figures from 2003 survey data from Kamadjeu et al (2006), data cited by Addo et al (2007) [12]

***WHO Global InfoBase, cited by Mensah (2008) [11]

****Ghana data from WHS, Cameroon data from STEPs Survey, cited by Kyobutungi (2008) [10]

To keep our discussion focused each country case study is presented under two headings: social knowledge of chronic diseases and their risk factors and; primary and secondary prevention strategies. For each case study the sets of questions outlined in our conceptual framework structured interpretation of available data.

## Results

### I Ghana

#### Social knowledge on chronic diseases and their risk factors

Chronic disease research in Ghana has traditionally been dominated by biomedicine and has focused primarily on the clinical aspects and medical adherence. More recently social science studies - mainly psychology and anthropology - have emerged that focus on knowledge, beliefs, representations and experiences of chronic diseases such as diabetes, hypertension, cancer and epilepsy [13,28], as well as studies on children with chronic diseases [29,30]. With few exceptions social science studies focus largely on southern urban communities. The local literature suggests that lay and patient knowledge of major chronic diseases is poor. Late presentations at medical facilities, healer-shopping (between biomedicine, ethnomedicine and faith healing) and poor self-care have been attributed to poor medical knowledge. For example women with breast cancer seek treatment at very late stages (3 and 4) at the Korle-Bu Teaching Hospital, due partly to poor knowledge of the condition: their survival rate is 25% [31]. Healer shopping within ethnomedical systems is reported to be common and is implicated in avoidable complications and deaths. However scientific and clinical work at the Centre for Scientific Research into Plant Medicine (CSRPM) suggests that effective ethnomedical drugs exist for arthritis, asthma, diabetes, hypertension and sickle-cell disease [32].

A dominant argument made in the regional literature is that chronic diseases are attributed to spiritual causes and that these spiritual causal theories inform lay engagement with traditional healing systems. However, a growing body of work in Ghana and other African countries suggest that chronic illness beliefs are rooted in complex socio-cultural knowledge systems. In a social psychological study of social representations of diabetes in rural and urban Ghana, de-Graft Aikins [13,33] identifies five sources from which rural and urban individuals draw knowledge on general health, pluralistic health systems, illness, chronic disease and diabetes: social (e.g family and friends), cultural (traditional handed-down knowledge), cross-cultural (through regional and international travel), institutions (pluralistic health professionals, mass media) and self (unique experiences of self in health and disease). These eclectic sources of knowledge inform multiple theories of diabetes which encompass diet (excessive sugar/starch), lifestyle, heredity, physiological disruption, contaminated foods and spiritual disruption (witchcraft and malevolent social actions). While individuals made spiritual causal attributions, the link between these attributions and healthcare choices was complex. First concepts of illness chronicity and incurability differ within cultures; in Ghana some ethnic groups such as the Akan accommodate chronicity [34], others like the Ga do not [28]. Secondly, concepts of medical pluralism are complex. Biomedical, ethnomedical and faith healing systems were subjected to public critique in terms of technical/practical knowledge of health problems, technological expertise, accessibility and ethics. All three systems had strengths and weaknesses across these criteria, depending on the health problem. People with diabetes engaged in nuanced legitimation processes when choosing practical information for diabetes care, especially with respect to pluralistic healthcare services. They engaged in four kinds of illness practices: biomedical management, spiritual action, cure-seeking and medical inaction. These forms of illness action highlighted the complex and unpredictable relationship between knowledge, beliefs and health seeking behaviours. Similar findings to the Ghanaian study are reported elsewhere in the region, including in Cameroon (see next) [15,35,36].

Research suggests that chronic disease knowledge is poor among health workers. Studies on diabetes highlight poor knowledge among doctors, nurses and conflicting knowledge among dieticians [28,30,37,42]. Studies on asthma highlight poor knowledge among junior doctors and general practitioners [38,39]. Cancer knowledge is poor among doctors and nurses [28]. Poor health worker knowledge has been implicated in poor communication, the development of complications and in healershopping [13,28,30]. Knowledge of chronic diseases is also poor within ethnomedical and faith healing systems, which provide a significant amount of healthcare, particularly in rural areas [13,40].

### Primary and secondary prevention strategies

#### Structural level

Four dimensions of structural responses have been identified in the global literature: policy, fiscal, engagement with industry and with international partners. Ghanaian responses have focused on policy and, to a lesser extent, engagement with industry (see next section).

Attempts were made to establish an NCD Control Programme in Ghana in the 1970s [41]. This followed the establishment of a Burkitt's lymphoma centre at KBTH in the mid-1960s and the development of a national cancer registry in the early 1970s. These early attempts faced operational, professional and political challenges. Formal discussion of Ghana's chronic disease burden resumed in the 1990s. Some conditions such as hypertension and diabetes were placed on the priority health intervention list of the Ministry of Health (MOH) [42,43]. A Non-communicable Disease Control Programme (NCDCP) was established in 1992, with an extensive remit for improving knowledge and advocacy for CVDs, diabetes, chronic respiratory diseases, cancers and sickle cell disease. In the last five years the NCDCP has convened national workshops on chronic diseases, advocated on radio, engaged in media training, advocated for tobacco control and participated in consultations towards alcohol policy development [41]. Despite these activities there is no policy or plan for chronic disease prevention. Local experts believe that chronic diseases are "neglected, constitute low policy priority and receive low interest from development partners" [41]. For instance, while the NCDCP is expected to play a public health role, it is poorly resourced and staffed entirely by medical professionals. However there have been other responses by the MOH to Ghana's health burden that are relevant to chronic disease prevention.

In 2006, the MOH implemented a National Health Insurance Scheme (NHIS), which includes medicines for hypertension, diabetes and some cancers on its exemption list. It is useful to note that the inclusion of some chronic disease medications have occurred as a result of lobbying by patient organisations (e.g. breast cancer) and research groups (e.g. sickle cell disease). Chronic disease care in Ghana is expensive. The monthly cost of treating conditions like diabetes exceeds the average salary [44]. For example, in 2007, the monthly cost of treating diabetes ranged between $106 and $638; the monthly cost for treating complications of diabetes (e.g dialysis for end-stage renal failure) was $1383 [44]. The minimum daily wage in 2007 was $2; the average monthly salary for a civil servant was $213 [44]. The financial burden of living with chronic disease exacerbates the psychosocial burden, for example it leads to family disruption and diminished family support. Studies suggest that the NHIS eases the financial burden of chronic disease for individuals able to afford the premium payments [28,30]. A Disability Bill was also introduced by the government in 2006. The Bill stipulates free access to general and specialist medical care for the disabled. Its significance for individuals disabled by chronic diseases (e.g impaired vision and limb amputations due to diabetic complications) has not been fully explored by interest groups.

#### Community level

Chronic disease prevention at community level should ideally encompass activities of the following key actors: primary health care services, voluntary organizations, the food industry and supermarkets, work sites, schools and the local media. In Ghana, the majority of these groups of actors have been involved in chronic disease prevention. We begin by documenting community level activities relevant to primary prevention and then focus on those relevant to secondary prevention.

Sedentary lifestyles have been strongly implicated in Ghana's chronic disease burden [45]. However there is also an emerging keep-fit culture in urban and rural areas. In the capital Accra and other major cities, a growing number of fitness centres offer physical fitness and general health services (e.g medical screening) [46]. Keep fit and football clubs are also common across the country; these clubs are usually run by, and dominated by, young men. The role of these organizations in promoting public health is important. However they cater to limited segments of society, such as the middle to high income urban middle class (for fitness centres) and to young men (for the keep fit clubs).

Churches, mosques and other faith-based institutions play an important role in health promotion. Churches have been visible facilitators of mass health walks, screening and health expert talks on public health problems. An estimated 65% of Ghana's population is Christian. Church members form strong civic ties within subgroups, such as the women's and men's fellowships or choirs. Research suggests that the church is an important source of information for lay people [34]; similarly people with chronic diseases rely on their churches for information and psychosocial support [13]. On the other hand religious institutions offer chronic disease treatment through their faith healing prayer camps or through Islamic divination. The impact of these practices is mixed. Research suggests that faith healing practices can cause disease complications for people with diabetes [13].

The mass media is a key site for disseminating information on chronic diseases in Ghana. Newspaper articles on cancer, sickle-cell disease, leukaemia, diabetes, hypertension and stroke appear in national publications such as the Daily Graphic and the Mirror, as well as their online versions. The local radio stations also tackle chronic diseases on their health programmes and present selected information on their websites (see for e.g. http://www.myjoyonline.com/radio/). Media information is either culled from international media sources or produced by local medical experts. Some experts write their own newspaper columns or host TV and radio shows. There is a growing trend of influential herbalists providing incorrect (chronic) disease information on radio and television as part of their advertising strategy.

Generally national newspaper coverage is low and few people read [47]. While radio has wider national coverage there is little knowledge of what is broadcast on rural radio. To address some of the challenges in media reportage the NCDP organised a training workshop for media representatives to increase media awareness, knowledge and reporting of chronic diseases [41]. The impact of this project is yet to be evaluated.

In 2005 the MOH established the Regenerative Health and Nutrition Programme (RHNP) which aimed to promote a preventative model of public health, rather than the dominant curative model [48]. The RHNP was not explicitly concerned with chronic disease, but its health enabling focus encompassed activities that reduce chronic disease risks, for instance eating more fruits and vegetables, reducing consumption of fatty foods and alcohol and taking up exercise. The programme was piloted in communities in eight regions through participatory education workshops. No baseline data was gathered on health knowledge or status prior to the programme, so it is difficult to evaluate the impact of the programme along these lines. However an independent review of the pilot programme [49] produced a number of insights: (1) the majority of programme recipients remembered key aspects of the nutrition and healthy lifestyles messages; (2) the easiest lifestyles to adopt were drinking more water and eating more fruits and vegetables, a challenging lifestyle was increasing physical activity, the most difficult was to reduce meat intake; (3) the high cost of fruit and vegetables in some regions and widespread perceptions of the toxicity of staple foods were barriers to adopting healthy lifestyles; (4) a minority of individuals had become advocates of the regenerative lifestyles; churches, mosques, the workplace and school were important spaces for advocacy. The pilot programme has not been replicated or scaled up. It has been commended as an important initiative for chronic disease prevention, but criticised for working in isolation from health services provided by the Ghana Health Service [48]. However, the RHNP is included in the MOH's current programme of work and it has entered a phase of engagement with industry and businesses through annual health fairs and public education via the mass media. A nutrition manual for schools and a strategic plan have been developed. These new developments are yet to be evaluated.

A number of patient advocacy groups exist for asthma, cancers (breast, leukaemia, prostate), diabetes, heart disease, hypertension and cardiovascular disease, epilepsy and kidney disease. Each organisation has different structures and modes of operation. The Korle-Bu Breast Cancer Clinic, Reach for Recovery, Mammocare and DWIB Leukemia Trust, provide support and advocacy services for individuals living with cancer. The Ghana Heart Foundation raises awareness on heart disease and provides clinical and surgical services for needy individuals with serious heart conditions. Basic Needs, an international mental health NGO provides education, psychosocial support and opportunities for enhancing livelihoods for people living with epilepsy http://www.basicneeds.org/ghana/. The Ghana Diabetes Association provides information and education on diabetes especially through World Diabetes Day events. Research suggests that advocacy groups help members to cope better with their conditions [13,28,30].

There are three major challenges in this area. The majority of advocacy services are located in the urban South and chiefly the capital Accra. This excludes a growing number of individuals living with chronic diseases in other parts of the country from accessing psychosocial support. The establishment of self-help groups in rural areas in the Brong Ahafo, Ashanti and Northern regions for example point to a need for national expansion of advocacy services ([13]; J. Adomako, pers communication, 2008). Second, with few exceptions, these services are run by healthcare professionals. Finally, while membership improves coping, there is no systematic information on how group membership and/or better coping improves self-care, management and health outcomes. There is growing evidence to suggest that patient-led self-help and advocacy groups have greater longevity and achieve more comprehensive sustainable goals (education, psychosocial support, advocacy) for their members [50,51]. Furthermore, research on sickle cell disease and chronic pain shows that skilled self-help groups can improve treatment and quality of life outcomes [51-53].

#### Individual level

At the individual level we focus on health service provision and individual pharmacological interventions. Medical facilities in Ghana are poorly equipped to treat chronic diseases: asthma, diabetes and sickle-cell disease are particularly affected by poor health services [13,28,30,39]. Challenges include poor infrastructure (both basic and sophisticated), inadequate training of healthcare providers (especially in terms of acquiring specialist knowledge of chronic conditions and of communicating knowledge to lay people and patients), and high cost of care. The challenges experienced by biomedical services are compounded by competing services provided by ethnomedical professionals and faith healers, which are unregulated, pharmacologically unsafe and are often implicated in avoidable complications [13]. There are few specialist chronic disease centres in the country. The country has only two specialist diabetes centres, situated in the two teaching hospitals in Accra and Kumasi, both southern urban cities. While general practitioners often run diabetes clinics in regional and district hospitals, they may lack the clinical depth of the specialist clinics.

Despite challenges to chronic disease treatment and management in Ghana, there is evidence of innovative care. The Korle-Bu Teaching Hospital's breast cancer clinic operates with a multidisciplinary team including surgeons, radiation oncologists, a clinical pharmacist and a clinical psychologist. This team works alongside cancer survivors (as peer supporters and counsellors) and a cancer advocacy group (Reach for Recovery). The clinic's approach has led to increased trust and improved communication between patients and health professionals [54] and created an important space for group education and psychosocial support [28].

### II Cameroon

#### Social knowledge of chronic diseases

Like Ghana, social science research on chronic diseases in Cameroon has emerged only in the last decade. Research has focused on diabetes, cancers and epilepsy and risk factors such as obesity and physical activity. There is a consensus that lay knowledge of chronic diseases is poor. Poor knowledge of chronic diseases leads patients and their carers to attribute these diseases to witchcraft and to initiate problematic treatment practices such as healer shopping within traditional healing systems [55]. This also impacts on patients and their carers' acceptance of and early engagement with biomedicine.

Awah et al (2008) reporting on an anthropological study of diabetes, observe that there is a lack of basic knowledge on diabetes and risk factors among people with diabetes [55]. This group often struggles to engage with biomedical treatment and management. Diet and weight management, which often involves weight loss, is one site of resistance. In Cameroon, as in many African societies, rapid weight loss is often attributed to HIV/AIDS status [56,57]. Thus Cameroonians with diabetes express fears about potential stigma they might experience from weight loss and a deviation from an accepted body size and social image [15]. The association of weight loss with HIV/AIDS stigma by people living with diabetes has been reported in the Ghanaian context [57].

Awah (2006) further observes a clash between expert and lay knowledge [58]. He notes that traditional knowledge stipulates that all diseases, including diabetes, can be cured. (This contrasts with some (Akan) traditional Ghanaian concepts of illness that accommodate the incurability and chronicity of some illnesses.) Health care professionals therefore have problems reconciling the biomedical emphasis on diabetes management with the traditional medicine emphasis on cure [15]. Yet deeper analysis of discursive constructions of diabetes suggests that causal attributions straddle the traditional and modern. A study of discourses on diabetes in Bafut, a rural village, shows that diabetes is referred to linguistically as *fumbgwuang *or *shugar*, often prefixed with *nighoni *(sickness, disease). *Nighoni-shugar *thus denotes 'sugar diseases' and *nighoni-fumbgwuang *'disease that is sweet'. Yet, *fumbgwuang *also refers to salt, indicating a taste that moves beyond the sweetness associated with sugar. Furthermore, traditional healers construct diabetes as a curse or a disciplinary agent, which is then used to call people to order and mete out justice. Thus, through discursive practices, diabetes straddles the traditional and modern; it has roots in modern lifestyles or is seen as a manifestation of a curse upon the family of the affected. This complex formulation, like the Ghanaian context, informs complex treatment choices, including healershopping, within the pluralistic medical sphere.

### Primary and Secondary Prevention

#### Structural Level

Cameroon is one of the few African countries that has developed a chronic disease policy focusing on diabetes and hypertension. The Health of Populations in Transit (HoPiT) team, a team of non-communicable chronic disease researchers in the Yaoundé University Teaching Hospital, in collaboration with the World Diabetes Foundation and the Cameroon Ministry of Public Health (MoPH), initiated the Cameroon Burden of Diabetes (CAMBoD) project. Research insights from the CAMBoD project led to the establishment of a programme of surveillance, prevention and control of diabetes and other chronic diseases, including cancer, epilepsy, sickle cell disease, deafness, stroke, and mental illness [59]. The MoPH created a Department for Disease Control (DDC) to monitor these diseases. Diabetes Clinics were established across the country with at least 18 diabetes clinics in Bamenda, Yaoundé and Douala and at least one clinic in each of the remaining regions. The CAMBoD project was also influential in reducing the prices of insulin and diabetes related products such as testing kits in across the country. For instance insulin was reduced from £15 to £3 [55]. The availability of generic drugs at subsidized rates and testing kits at reduced prices is an important step in secondary prevention. While the Cameroonian government has made important strides in diabetes care, especially in its commitment to providing quality health services, challenges exist. Community involvement in the prevention and treatment of major chronic diseases is still low (see next). Also, although policies exist, their implementation is problematic [55]. The HoPiT team together with the MoPH's Department for Disease Control have been involved in a number of prevention activities including organising training workshops for health personnel, carrying out STEPwise surveys to identify the risk factors for common chronic diseases in both urban and rural areas and providing monitoring services of chronic diseases.

#### Community Level

Faith based organisations and chronic disease advocacy groups play some role in chronic disease prevention in Cameroon. Health centres tend to provide the majority of support. Unlike Ghana fitness centres and the mass media do not play a significant role in chronic disease prevention.

Religious institutions such as churches often focus on a limited number of chronic diseases. For example, the Full Gospel Church - a Pentecostal church which has branches nationwide - offers compulsory pre-nuptial exams on sickle-cell disease and HIV/AIDS to identify couples' risk status. The church also invites health experts to provide advice on hypertension and diabetes. The Presbyterian and Catholic churches include health awareness information in the yearly study materials they provide to their women and men's groups. Chronic diseases such as HIV/AIDS, diabetes, hypertension, cancers and epilepsy receive a fare share of the lessons especially in terms of prevention and support of the sick.

While the fitness industry and the mass media do not play as significant a role in chronic disease prevention as is reported in Ghana, it is worth commenting on the available information. In the early 1990s the Cameroon government created fitness tracks, termed 'parcours vitas'. These were created in most of the provincial headquarters to increase the activity levels of its citizens. The tracks had facilities for different exercises. Due to poor management, these tracks deteriorated and have been abandoned. Most fitness centres are private, expensive and tend to be elitist. Most urban dwellers do not have access to these centres. Media coverage on chronic diseases in Cameroon is minimal compared to that of communicable diseases such as malaria and HIV/AIDS. However, the HoPiT programme coverage in health institutions and public places provides extensive information on hypertension and diabetes. Billboards advertising cigarettes also warn on the dangers of smoking in relation to cancer.

In the capital Yaoundé there are advocacy organisations for cancers. The Cameroon National Fight against Cancer organises screenings of prostate and cervical cancers twice every year. The Cameroon Baptist Church Health Board Cervical Cancer and Women's Health Program also launched a mobile cervical cancer screening clinic using a US-donated military ambulance. There are no community support groups for cancer patients.

There are no psychosocial support or advocacy services for people living with chronic diseases outside of the capital. This contrasts sharply with community-based support groups for infectious diseases such as HIV/AIDS. Health facilities tend to offer the majority of support services. The services provided by health facilities are supported by the HoPiT team who provide educational flyers on diabetes and education centres in hospitals [58]. There are no psychosocial support and advocacy services for asthma, epilepsy and sickle cell disease.

#### Individual Level

There are diabetes and hypertensive clinics in all the regions of the country. These clinics are responsible for screening, treatment and public education. The National Cancer Board also carries out bi-annual free screening exercises on breast, cervical and prostate cancers at the General Hospitals of Douala and Yaoundé. Teams are also sent out to the different regions of the country twice a year on a yearly basis. However challenges exist. Most health facilities especially in rural areas are ill-equipped to deal with chronic diseases such as sickle cell, cancers and diabetes. Health care workers are also not well trained to provide public health education on risk factors and to provide effective treatment. There is a strong link between training health workers on chronic disease management and improvement in quality of care. In rural Bafut, a nurse led care initiative for epilepsy resulted in significant drop in the number of seizures [60]. This approach has been piloted in other African countries including Kenya [61] Tanzania [62] and Malawi [63].

## Discussion

Ghana and Cameroon share similarities on their chronic disease burden. Prevalence rates for hypertension are high in both countries. Risk factors for major chronic conditions, such as high prevalence of overweight and obesity and low physical activity levels, are similar. There are also similarities in terms of the gendered and class-based nature of prevalence and risk factors. In both countries obesity levels are higher among women, smoking prevalence and alcohol consumption is higher among men, and physical activity is lower among urban communities [45,64].

Issues around knowledge, self-care and management are similar in both countries. Medical knowledge is poor and engagement with biomedical services is poor. Studies report late engagement with biomedical care (e.g for Ghanaian women with breast cancer, for people with diabetes in both countries) and ideological clashes between lay and expert groups (in Cameroon): these lead to avoidable complications, disability and death. However social knowledge on causes and treatment of chronic diseases are complex and this shapes complex unpredictable engagement with pluralistic health systems. Research suggests intra-cultural differences across important conceptual issues on chronic disease risk and treatment. In Ghana there are ethnic differences on food practices and on concepts of illness chronicity. Studies on diabetes attributions and experiences in both countries demonstrate that local systems of knowledge (social logic) transcend the restricted system of biomedical knowledge (medical logic). Deeper analysis highlights areas of conceptual and practical convergence between medical and social logic. These areas of convergence provide important opportunities for developing effective secondary prevention.

However there are fundamental differences between Ghana and Cameroon in terms of 'multi-institutional and multi-faceted responses' to their chronic disease burden (see Table 4).

**Table 4:** Chronic Disease Prevention Activities in Ghana and Cameroon

	Ghana	Cameroon
**Structural**		

Policy	**++**	**+++**

Fiscal	-	-

Industry and Business	-	-

International collaboration	**+**	**+++**

**Community**		

Mass media	**++**	**+**

Voluntary/advocacy organisations	**+++**	**+**

Institutions (school, workplace, churches)	**++**	**+**

Primary healthcare	**+**	**++**

**Individual**		

Lifestyle	**+**	**+**

Pharmacological interventions	**+**	**++**

**Key**: +++ (ideal, compared to existing good practices); ++ (satisfactory); + (poor); - (strategy not available)

In Ghana there is a significant gap between policy rhetoric and action. Despite almost two decades of policy discussions on the need for a chronic disease policy, there is no concrete policy or plan. Although a non-communicable disease control programme has been established which advocates a public health model, the programme lacks the professional and material capacity to achieve its goals. However Ghana has established a National Health Insurance Scheme that covers treatment of some chronic diseases, a disability bill has been passed which may benefit individuals disabled by chronic diseases, and there is a strong presence of community-based action through patient advocacy groups which receive support from the medical community. There is also a culture of media reportage on chronic diseases with strong reliance on medical experts. It can be argued that Ghana's response is multi-institutional and multi-faceted, although diverse institutional responses remain to be integrated, critically evaluated, formalised and incorporated into policy development. A bottom-up approach dominates the Ghanaian chronic disease arena.

In Cameroon a chronic disease policy has been developed and implemented. This policy commands concrete structural investment influenced by committed research and donor communities [55]. Diabetes and hypertension clinics have been established across the country as a result of this policy, there is concrete research-led health worker training and patient education at these centres, and the cost of drugs and disease management products have been reduced. Furthermore, advocacy organisations based largely in the capital Yaounde provide education on diabetes, cancers and chronic disease risk factors such as smoking. However challenges exist. There have been problems with policy implementation and in translating health worker training into improved quality of care. A clash between expert and lay knowledge is reported. There appears to be low community engagement with chronic disease issues, there are fewer advocacy organisations and they focus solely on education and not patient support. The mass media neglects chronic diseases. A top-down approach dominates the Cameroonian chronic disease arena.

In both contexts it is evident that a focus on bottom-up or top-down approach solely will not be sustainable for nationwide inclusive chronic disease prevention. As the social psychology of participation approach suggests a multi-level framework that encompasses top-down and bottom up responses will offer a more successful model for prevention. For example without structural investment the activities of cash-strapped advocacy organisations in Ghana will remain at the level of southern urban cities. And without active incorporation of lay perspectives, experiences and practices, the Cameroonian policies will not have a behavioural impact on the population and on patients. How do both countries move towards a multi-level framework of chronic disease prevention? We argue that each country offers practical insights for the other to draw on. We focus on three areas of knowledge transfer: (1) policy development; (2) active incorporation of lay perspectives in primary and secondary intervention or 'participatory chronic disease prevention'; and (3) investment in local and indigenous 'knowledge brokers'.

### Policy development

Ghanaian health policymakers could benefit from a HoPIT style multidisciplinary research culture that is committed to applied research and advocacy, attracts funding from external donors and is skilled at managing the local politics of policymaking [55]. The regional neglect of chronic disease is partly due to the politics of international health funding and partly due to leadership and governance challenges. It is well documented that the development of local health policies is often influenced by the priorities and ideologies of international health initiatives and Africa's development partners [65]. These factors are often compounded by poor leadership and governance [66]. The establishment of the health Millennium Development Goals (MDGs) as a gold standard of measuring outcomes in global population health and of international funding and monitoring initiatives such as the Global Fund is a recent case in point. In many African countries, health policymakers prioritise public health problems identified by the health MDGs such as HIV/AIDS, tuberculosis and malaria and neglect important public health problems that are not explicitly mentioned by the health MDGs, even when prevalence, morbidity and mortality rates from the latter group far outweigh that of the former group. Ghana's policy response to its chronic disease burden falls under this category.

Cameroon appears to have escaped this policy challenge and developed a priority-based approach that accommodates policy on communicable diseases and chronic diseases. The Cameroonian model has two key strengths. First, the HoPIT research team is multidisciplinary; medical and social scientists conceptualise, develop and implement national interventions. In Ghana the cumulative efforts of researchers may be termed multidisciplinary, but there are as yet no multidisciplinary teams directing theory, practice and policy on a community-based chronic disease programme. Second, the team invests in bold leadership, creative management of local politics of health administration and policymaking and strategic networking and collaboration with international partners.

### Participatory chronic disease prevention

Ghanaian and Cameroonian responses differ in terms of community and patient involvement in chronic disease prevention. There are more patient and advocacy groups in Ghana than there are in Cameroon. Their focus and mode of operation falls within the bottom-up approach of health promotion. The Ghanaian responses have evolved organically through shared illness experiences and the frustrations of medical professionals working under difficult circumstances. However the nature of the responses - education and psychosocial support - lends itself to systematic development of primary and secondary prevention strategies that are aligned to current ideas on empowering communities to engage in healthy lifestyles and practices [67] and to investing in 'patient partnerships' [68]. We identified three key challenges in Ghana that must inform future research on the effectiveness of advocacy organisations: (1) establishing advocacy organisations beyond the nation's capital; (2) supporting the development of patient-led advocacy groups; (3) evaluating the impact of advocacy activities on self-care and health outcomes. Generally there is strong evidence of complex lay knowledge on health, illness and chronic diseases in both countries that must inform community-based interventions. In terms of population-based primary prevention, education and advocacy strategies must take into account the differences in prevalence rates across gender, socio-economic status and geographical location and the evidence on ethnic differences in chronic disease concepts.

### Investing in local Knowledge Brokers

A growing global call for incorporating chronic diseases - such as CVD, respiratory diseases and diabetes - more explicitly into the MDGs might change the course of regional policy responses [69]. Until then it is fair to say that, due to competing interests, a significant gap will remain between chronic disease policy rhetoric and practice in sub-Saharan Africa. Practical responses to the continent's chronic disease epidemic will have pay close attention to cost-effective interventions that reach national populations. Literature highlights the mass media as the most cost-effective form of chronic disease intervention; community spaces with effective opinion leaders have also been singled out for attention [6-8]. In both countries faith-based organisations, in particular churches, and the mass media were identified as important 'knowledge brokers' in this regard. Their activities need to be appropriately documented, analysed and incorporated into chronic disease policy development.

In both countries churches constitute important spaces for health education and advocacy. Some focus on chronic disease education and screening. Their successful organically driven pastoral activities can be formally harnessed for national level chronic disease prevention initiatives. The role of churches in HIVAIDS prevention offer useful models for chronic disease prevention [70,71]. However the potentially damaging treatment repertoires of faith healers and prayer camps will require policy attention.

Although both countries have a culture of media health reporting, Ghana has a stronger chronic disease media reportage culture than Cameroon. The Ghanaian approach will be a useful model although it requires critical development along at least three lines: (1) regulating sources of public health information; (2) including experts (both professional and lay) who can provide 'effective' information on the cultural and behavioural dimensions of primary and secondary prevention; and (3) training journalists in chronic disease reporting along the lines of media reportage in high income countries like the UK and the US.

Generally, for both countries, it will be important to identify what kinds of media work for chronic disease education. In Africa radio is an important space for knowledge dissemination. Ghana has 84 radio stations; 68.91% of the population listen to radio once a day. Cameroon has 61 radio stations; 51.9% listen to radio once a day [47]. To a lesser extent television and newspapers have been seen as a major source of health advice along with billboard messaging. The emergence of glossy magazines [72] along with uptake of new technologies - SMS messaging via mobile phones and to a lesser extent the internet are also being seen as a future source of healthcare information delivery for Africa [72].

Frequently programmes are assessed as effective if they have high audience figures, although they may not always be useful to or appropriate for audiences [73]. Assessing the impact of media campaigns, particularly in rural and poorer areas is very difficult to achieve [73]. The content of messaging is often influenced by funding and it appears that many media initiatives from problem pages through to advertorials are not evaluated critically in terms of impact and effectiveness. Moreover, the complexity of campaigns and different delivery systems (for example messages run concurrently across television, radio and billboards) can make it difficult to assess what information people find most accessible and useful.

## Conclusion

Chronic diseases present complex medical, psychosocial, economic and political challenges in Africa. These challenges undermine the development of effective and sustainable primary and secondary interventions. We have demonstrated that two low-income countries struggling to contain a double burden of disease with minimal financial and human resources have developed practical responses to their chronic disease burden. Some of these responses can be classed as 'good practice' as they constitute replicable models of primary and secondary prevention described in the literature. However it is difficult to measure the extent to which these initiatives improve health outcomes or are cost-effective. Health financing initiatives have eased the economic burden of chronic disease in both countries for some patients. But the rising cost on healthcare systems and budgets has not been documented or evaluated. In Ghana, people with diabetes and cancers state the importance of patient advocacy groups on their ability to cope with the psychosocial burden of disease; but in both countries there have been no systematic studies on the impact of self-help groups in improving self-care, coping, and general health outcomes. Without evaluation, important interventions cannot be replicated or scaled up. Intervention and cost-effectiveness studies will be useful additions to future research. There are best practice models within Africa: for example South Africa's fiscal policy on tobacco and its impact on tobacco use [1]; structural and community-based intervention strategies in Mauritius that led to the adoption of healthier diets and reduced cholesterol levels [8]; and a recent study in Burkina Faso that examined the catastrophic (economic) consequences of living with chronic disease across economic status, household characteristics and illness profiles [74]. Outside Africa, robust intervention models exist in Finland, the UK and the US [1]. These models should guide future development of sustainable primary and secondary interventions in Ghana and Cameroon.

## Competing interests

The authors declare that they have no competing interests.

## Authors' contributions

ADGA and PB conceived of the topic and the conceptual framework. ADGA drafted the manuscript and contributed the information on Ghana. LLA contributed the information on Cameroon. PB contributed information on media and health promotion in Africa. All authors contributed to data synthesis and read and approved the final manuscript.

## Authors' information

ADGA is a research fellow in social psychology at the University of Cambridge. Her research focuses on representations and experiences of chronic physical and mental illnesses among African communities on the continent and in the UK. She is currently principal investigator of the UK-Africa Academic Partnership on Chronic Disease, a project funded by the British Academy.

PB is a social psychologist based at University College London where she lectures on an MSc in International Primary Health Care. She specialises in research on media messaging and health and applies her research and education through advice giving in the media and training journalists to deliver healthcare messages.

LLA is a lecturer of Gender and Discourse Studies at the University of Dschang. She holds a PhD from Lancaster University in gender and language. She is currently specialising in lay medical discourse with an emphasis on how chronic diseases are discursively represented in Africa and the resulting implications on prevention.
